# CCN Family Member 2/Connective Tissue Growth Factor (CCN2/CTGF) Has Anti-Aging Effects That Protect Articular Cartilage from Age-Related Degenerative Changes

**DOI:** 10.1371/journal.pone.0071156

**Published:** 2013-08-12

**Authors:** Shinsuke Itoh, Takako Hattori, Nao Tomita, Eriko Aoyama, Yasutaka Yutani, Takashi Yamashiro, Masaharu Takigawa

**Affiliations:** 1 Department of Biochemistry and Molecular Dentistry, Okayama University Graduate School of Medicine, Dentistry, and Pharmaceutical Sciences, Okayama, Japan; 2 Department of Orthodontics, Okayama University Graduate School of Medicine, Dentistry, and Pharmaceutical Sciences, Okayama, Japan; 3 Biodental Research Center, Okayama University Dental School, Okayama, Japan; 4 Yutani Orthopaedic Clinic, Kobe, Japan; The University of Hong Kong, Hong Kong

## Abstract

To examine the role of connective tissue growth factor CCN2/CTGF (CCN2) in the maintenance of the articular cartilaginous phenotype, we analyzed knee joints from aging transgenic mice (TG) overexpressing CCN2 driven by the *Col2a1* promoter. Knee joints from 3-, 14-, 40-, and 60-day-old and 5-, 12-, 18-, 21-, and 24-month-old littermates were analyzed. *Ccn2-LacZ* transgene expression in articular cartilage was followed by X-gal staining until 5 months of age. Overexpression of CCN2 protein was confirmed through all ages in TG articular cartilage and in growth plates. Radiographic analysis of knee joints showed a narrowing joint space and other features of osteoarthritis in 50% of WT, but not in any of the TG mice. Transgenic articular cartilage showed enhanced toluidine blue and safranin-O staining as well as chondrocyte proliferation but reduced staining for type X and I collagen and MMP-13 as compared with those parameters for WT cartilage. Staining for aggrecan neoepitope, a marker of aggrecan degradation in WT articular cartilage, increased at 5 and 12 months, but disappeared at 24 months due to loss of cartilage; whereas it was reduced in TG articular cartilage after 12 months. Expression of cartilage genes and MMPs under cyclic tension stress (CTS) was measured by using primary cultures of chondrocytes obtained from wild-type (WT) rib cartilage and TG or WT epiphyseal cartilage. CTS applied to primary cultures of mock-transfected rib chondrocytes from WT cartilage and WT epiphyseal cartilage induced expression of *Col1a1*, *ColXa1*, *Mmp-13*, and *Mmp-9* mRNAs; however, their levels were not affected in CCN2-overexpressing chondrocytes and TG epiphyseal cartilage. In conclusion, cartilage-specific overexpression of CCN2 during the developmental and growth periods reduced age-related changes in articular cartilage. Thus CCN2 may play a role as an anti-aging factor by stabilizing articular cartilage.

## Introduction

CCN2/CTGF (CCN family member 2/connective tissue growth factor, CCN2) is a cartilage-maintaining protein that is dominantly expressed in cartilage; and it strongly enhances the production of cartilaginous matrix proteins, such as type II collagen (*Col2a1*) and aggrecan, as well as stimulates chondrocyte proliferation, differentiation and maturation of growth-plate chondrocytes under physiological conditions [1], [2], [3], [4], [5]. Although CCN2 stimulates the proliferation and differentiation of various types of chondrocytes, it does not stimulate hypertrophy of articular and auricular chondrocytes [6], [7]. CCN2 also enhances the adhesion of chondrocytes to fibronectin through integrin [8] and angiogenesis by enhancing adhesion and migration of endothelial cells *in vivo* [3], [9]. CCN2-deficient mice show skeletal dysmorphism as a result of impaired chondrocyte proliferation and extracellular matrix composition within the hypertrophic zone, indicating that CCN2 is a crucial regulator of extracellular cartilage matrix formation [10]. Furthermore, implantation of CCN2-incorporated gelatin hydrogel into full-thickness defects of rat articular cartilage accelerate cartilage repair [11].

This raises the question as to whether, and if so, to what extent CCN2 is involved in the maintenance of the chondrocyte phenotype and protection from degenerative changes in aging cartilage or in osteoarthritis (OA). Multiple factors may cause OA, the most common of all joint disorders and showing increased incidence with age, the symptoms of which include genetic/age-related alterations in extracellular matrix (EM) components, biomechanical stress or an imbalance in synovial homeostasis [12], [13]. The disease is characterized by breakdown of the cartilage matrix followed by development of surface fibrillations and fissures, and these changes can lead ultimately to complete loss of articular cartilage. Another characteristic of OA is hypertrophy and ectopic growth of bony structures in the joints. Thickening of the subchondral bone combined with loss of articular cartilage leads to increased stiffness and reduced shock-absorbing capacity of the bone [14], [15].

Understanding the basic mechanisms by which aging affects joint tissues may also help to unravel new targets for treatment or prevention of OA. Currently, there are no reliable pharmacological agents able to prevent or even reverse degeneration of articular cartilage occurring in OA. In animal models, osteoarthritis-like changes can be initiated by application of proteinases such as matrix metalloproteinases (MMPs) and aggrecanases, which digest type II collagen (COL2) and proteoglycan [16], [17], the principal components of the matrix of articular cartilage. However, trials applying proteinase inhibitors for clinical use as a disease-modifying treatment have to date been unsuccessful because of insufficient efficacy and severely adverse side effects [18], [19], thus turning the interest of researchers to the upstream signals of the proteinases in chondrocytes.

Joint cartilage is a permanent cartilage not destined to be replaced by bone, unlike growth-plate cartilage, but recently evidence is accumulating showing that in the late stages of osteoarthritis, articular chondrocytes undergo a differentiation process similar to that occurring during endochondral ossification [20], [21]. Several studies have shown the expression of type X collagen (COL10) in latestage osteoarthritic cartilage [22], [23]; also, the expression of other markers of chondrocyte hypertrophy, such as matrix metalloproteinase (MMP)-13 [24], annexin VI [25], alkaline phosphatase [25], [26], osteopontin [27], and osteocalcin [26] are enhanced in osteoarthritic cartilage, as well as markers of chondrocyte dedifferentiation such as type I collagen (*Col1a1*) [28], [29]. These findings indicate that in the case of osteoarthritis articular chondrocytes do not maintain a stable phenotype and lose their characteristics of permanent cartilage.

In order to elucidate the role of CCN2 during chondrogenesis, cartilage maturation, and stabilization of the chondrocyte phenotype in articular cartilage, we generated mice overexpressing *Ccn2* under the control of the *Col2a1* promoter to clarify the role of CCN2 in chondrogenesis and skeletogenesis, as well as in adult cartilage. During the embryonic stage and growth period, overexpression of CCN2 enhances chondrocyte proliferation and the production of extracellular matrices through the induction of IGF-I and II, resulting in enhanced endochondral bone formation and extended bone length. Since these mice show strong accumulation of extracellular matrix in all of their cartilages [5], we hypothesized that overexpression of CCN2 may have an effect on adult cartilage by conferring resistance to age-related degenerative changes in joints. In the present study, we analyzed knee joints from littermates of aged CCN2 TG mice and WT controls. Our findings indicate that cartilage-specific overexpression of CCN2 stabilized the phenotype of articular chondrocytes in aging mice by enhancing the synthesis of aggrecan, while suppressing chondrocyte dedifferentiation and hypertrophy.

## Materials and Methods

### Animals

For overexpression of CCN2 in cartilage, HA-tagged *Ccn2* cDNA and *IRES-LacZ* as an expression marker were cloned for expression under the control of a 6-kb *Col2a1* promoter-enhancer (for details, please see [5]). Littermates of 3-, 14-, 40-, and 60-day-old and 5-month-old (1 male TG and 1 male WT), 12-month-old (2 male TG and 2 male WT), 18- 21-, and 24-month-old (2 male TG and 1 male WT) mice were used for analysis of the knee joints. 21-month-old (5 TG, 1 male and 4 females; and 1 male WT) and 18-month-old (3 WT males and 2 WT females) mice were used to analyze statistically the conditions of the articular cartilage of their knee joints. Knee joints of sacrificed mice were isolated for histology. All mice were housed in filter-top cages with paper-chip bedding under standard pathogen-free conditions. They were fed a standard diet with tap water provided *ad libitum*.

### Ethics Statement

Experiments were performed according to the Animal Ethics Committee of the Okayama University (permission #09035, 08002, 11034 for DNA recombination experiments, #OKU-2012113 for animal experiments).

### X-gal Staining

For confirmation of the expression of the transgene in articular cartilage, LacZ activity of 14-, 40-, 60-, and 150-day-old transgenic and wild-type mice was detected by staining with X-gal (5-bromo-4-chloro-3-indolyl-D-galactopyranoside; Roche) overnight following fixation in phosphate-buffered glutaraldehyde and formaldehyde for 1 hour, as described before [8], [30]. For staining of knee joints, the skin and muscles were removed before fixation. X-gal-stained knee joints were postfixed overnight in 4% formaldehyde, dehydrated, and embedded in paraffin. Seven-micrometer-thick sections were prepared by standard methods and counterstained with eosin.

### Radiographic Analysis

Degenerative changes in knee joints were analyzed by inspecting soft-x-ray radiographs. The legs were dissected at proximal side of the femur immediately after sacrificing the mice to avoid external movement of the joint. Images of natural flexion position of knee joints from the lateral side were obtained under consistent conditions (40 kV, 5 mA for 3 sec; Fujicolor, Sofron SRO-M50, Tokyo, Japan). Diagnostic analysis from radiographs of knee joints was done according to previous studies on osteoarthritic changes in STR/Ort mice [31], [32], [33], [34].

### Histology

Knee joints were dissected and fixed in 4% phosphate-buffered formaldehyde for 24 hours. Following fixation, the samples were defatted by passage through a series of ethanol and decalcified in 0.5 M EDTA for 3 weeks. After the tissues had been dehydrated and embedded in paraffin, serial frontal sections of 7-µm-thickness were cut through the knee joints. The sections were first stained with Safranin-O-fast green [35] for detection of proteoglycans and for measuring the thickness of the cartilage layer in the knee joints. For toluidine blue staining, they were then deparaffinized and rehydrated, stained with 0.1% toluidine blue for 3 minutes, and washed in running tap water for 2 minutes. The slides were then dehydrated and mounted with Mountquick (Daido Sangyo, Tokyo, Japan).

### Immunohistochemistry

Immunohistochemistry was done as reported previously [8]. Sections of knee joints were treated with bovine testicular hyaluronidase (25 mg/ml) for 30 minutes at room temperature for epitope retrieval and then immunostained with anti-CCN2 (Abcam, Cambridge, UK), anti-aggrecan neoepitope (Novus Biologicals, Littleton, CO), anti-type I (Millipore, Billerica, MA), type II (CII D3, [36]), and type X collagen (X53, both kindly provided by Dr. von der Mark, University of Erlangen) and anti-MMP13 antibodies (Millipore). Cell proliferation analysis was performed by using a PCNA staining kit (Invitrogen, Carlsbad, CA) according to the manufacturer’s protocol.

### Image Analysis and Statistical Analysis

The proteoglycan content and staining intensity of CCN2, PCNA, and type I, type II and Type X collagens were assessed by encircling the stained area; and mean of the staining intensity in the whole area of the medial tibial cartilage was measured with a computerized imaging system (AxioVision, ZEISS, Oberkochen, Germany). The average intensity between the TG and WT mice cartilage was analyzed statistically by using Student’s t-test.

For semiquantitative assessment of MMP-13 levels in the articular cartilage, the total number of chondrocytes in equal fields of load-bearing regions in the medial portion of the tibial cartilage of TG and WT animals was counted, and MMP-13 levels in the counted chondrocytes were measured densitometrically by a computerized imaging system after staining with specific antibodies. For the staining intensity of MMP-13, we measured luminescence by using Axiovision fluorescence software, and staining intensity was counted as dark (low) luminescence. All of the extracted cells were pooled, and average distribution of luminescence of TG and WT groups was compared statistically by use of the chi (χ)- square test.

### Cyclic Tension Stress (CTS)

For transfection with the CCN2-overexpression vector, primary cultures of growth-plate chondrocytes were prepared from the ribs of newborn WT mice by use of collagenase treatment [8], [37]. After transfection with pEGFP/CCN2 or pEGFP DNA by electroporation, the cells (1× 10^6^ cells/chamber) were plated in Flexible chambers (STREX, Osaka, Japan) that had been pre-coated with 2 ml of 0.05 mg/ml fibronectin. For preparation of primary cultures of articular chondrocytes from 6-day-old TG and WT mice, the upper 2/3 part of epiphyseal cartilage (without the growth plate) of elbow, shoulder, hip, and knee joints were taken and treated with collagenase. The cells from TG and WT were separately pooled and cultured in alpha-MEM (MP Biomedicals, Solon, OH) containing 10% FCS and 50 µg/ml of ascorbic acid. After the cells had reached confluence, the medium was changed; and after a 24-hour incubation the chambers were moved to the Flex cell culture system (STREX, STB-140). A cyclic tension load of stress at 0.5 Hz, with 6% elongation was applied for 12 hours. Then the cells were harvested for RNA preparation according to the manufacturer’s instructions (RNeasy mini kit, QIAGEN, Hilden, Germany). Control chambers were treated in a same way but without CTS.

Reverse transcription was carried out by using avian myeloblastosis virus (AMV) reverse transcriptase with 300 ng of each total RNA according to the manufacturer’s protocol (Takara Bio, Ohtsu, Japan). Quantitative PCR was carried out by using the SYBR-Green PCR assay (Toyobo, Osaka, Japan) and specific primers, with 2 replicates. Experiments were performed with a StepOnePlus Real-Time PCR System (Applied Biosystems, Foster City, CA). The PCR cycling conditions were set to 10 min at 95°C followed by 50 cycles of 30 s at 60°C, 40 s at 72°C, and 30 s at 95°C. One cDNA sample, which gave the highest copy number for each primer set, was diluted to generating standard curves for relative quantification of other samples. The expression level was standardized to Gapdh. The results were evaluated statistically by using Student’s t-test. Primers used for RT-PCR were the following: 5′-ctccacccgagttaccaatgacaa-3′ and 5′-ccagaaagctcaaacttgacaggc-3′ for *Ccn2*; 5′-tggtggagcagcaagagcaa-3′ and 5′-cagtggacagtagacggaggaaa-3′ for *Col2a1*; 5′-cagggttcccagtgttcagt-3′ and 5′-ctgctcccagtctcaactcc-3′ for *Aggrecan*; 5′-tgctgcctcaaataccctttct-3′ and 5′-tggcgtatgggatgaagtattg-3′ for *ColXa1*; 5′-agttggtgctaagggtgaag-3′ and 5′-gcaataccaggagcaccatt-3′ for *Col1a1*; 5′-tcctcggagactggtaatgg-3′ and 5′-tgatgaaacctggacaagca-3′ for *Mmp-13*; 5′-caatccttgcaatgtggatg-3′ and 5′-taaggaaggggccctgtaat-3′ for *Mmp-9*; and 5′-caatgaccccttcattgacc-3′ and 5′-gacaagcttcccgttctcag-3′ for *Gapdh*. For overexpression of GFP-CCN2 in WT rib-cage chondrocytes, the chondrocytes were pooled from 6 to 7 embryos; and for preparation of articular chondrocytes from TG and WT epiphyseal cartilage, the articular chondrocytes were pooled from 2–3 WTs or TGs. Two different CTS experiments for each cell preparation were done, and the cells were prepared 3 times and showed similar effects. The most typical results were shown in the Figures.

## Results

### Overexpression of CCN2 in Articular Cartilage of Transgenic Mice

**Figure 1:**
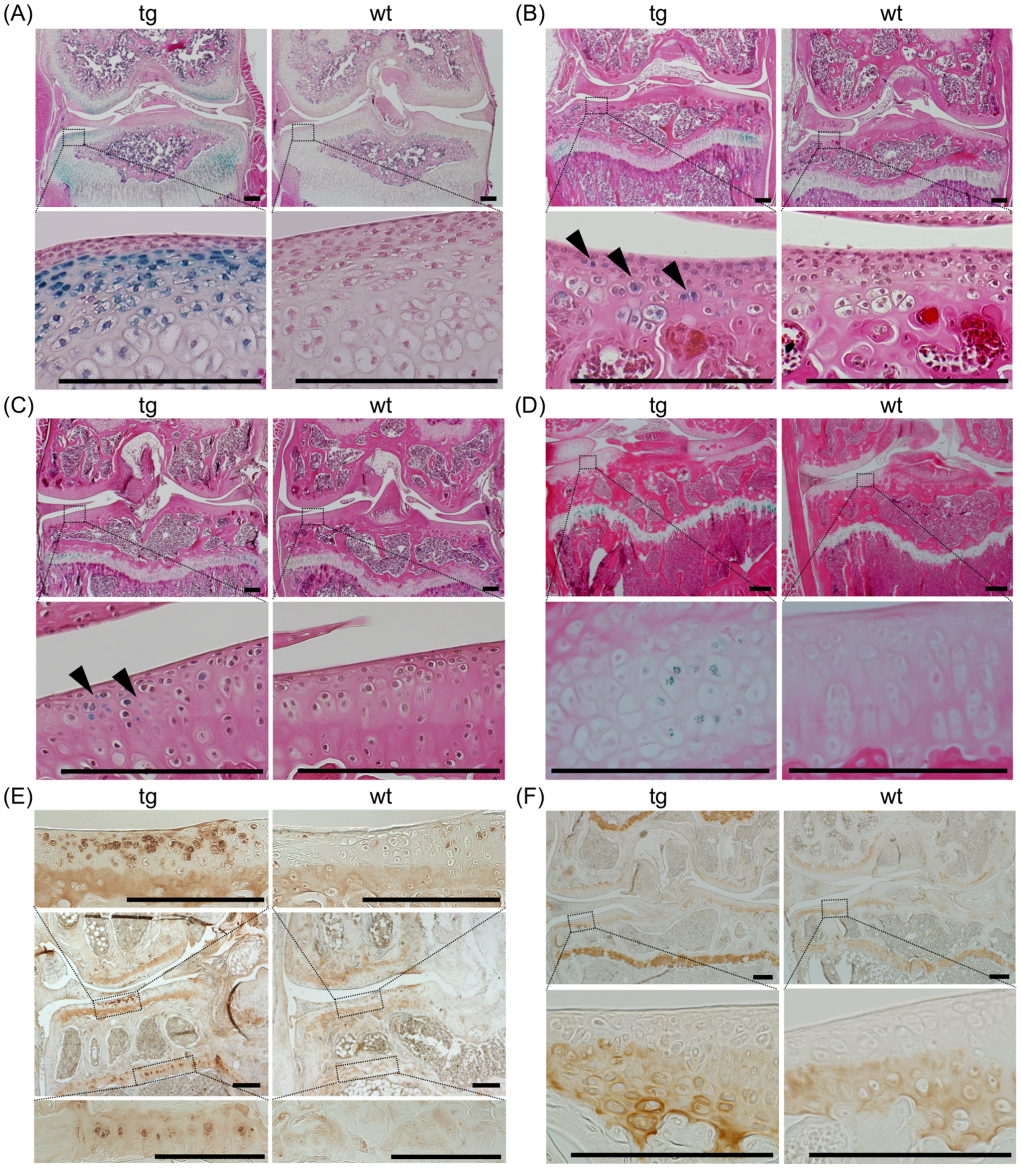
Overexpression of CCN2 and accumulation of type II collagen in aged articular cartilage of transgenic mice. (A–D) Frontal sections of knee joints after X-gal staining. Specimens from 14- (A), 40- (B), 60- (C), and 150-day-old (D) TG (left) and WT (right) mice. Lower panels show magnified regions of tibial articular cartilage as indicated by the dotted boxes in the upper panels. Expression of *LacZ* in the articular cartilage was detected in the TG sections. Arrowheads indicate *LacZ-*positive articular chondrocytes. Bars: 200 µm. (E) Immunohistochemical staining of CCN2 in frontal sections of knee joints from 21-month-old TG and WT mice. Upper and lower panels show magnified load-bearing regions of tibial articular cartilage (upper) and growth-plate cartilage (lower), as indicated by the dotted boxes in the middle panels. Bars: 200 µm. Deposition of CCN2 in the upper zone of TG articular cartilage was enhanced compared with that in WT articular cartilage. (F) Immunohistochemical staining of type II collagen in frontal sections of knee joint from 21-month-old TG and WT mice. Lower panels show magnified load-bearing regions of tibial articular cartilage, as indicated by the dotted boxes in the upper panels. Bars: 200 µm. Accumulation of type II collagen is enhanced in TG growth-plate cartilage as compared with WT, but in articular cartilage the difference was not significant.

We first examined whether and how long the expression of the *Ccn2-LacZ* transgene under the control of the *Col2a1* promoter would continue to be expressed in articular cartilage of transgenic mice after birth. X-gal-positive cells were detected in the articular cartilage and growth plates of 14-day-old TG animals (Figure 1A). In specimens of articular cartilage from 40-, 60- and 150-day-old animals, X-gal-positive chondrocytes were reduced in number, but still clearly detectable (Figure 1B, C, and D). This reduction in transgene expression in articular cartilage with age is consistent with the finding that adult articular chondrocytes express very low levels of type II collagen [38].

In contrast, the immunohistochemical analysis revealed significantly enhanced accumulation of CCN2 protein in growth-plate cartilage in the superficial and deep zones of articular cartilage of knee joints from 21-month-old TG mice (Figure 1E, Figure S1F); although the number of X-gal-positive cells was lower in 5-month-old mice (Figure 1D), suggesting that overexpressed CCN2 had stably accumulated in the extracellular matrices. To estimate the overaccumulation of CCN2 in the TG articular cartilage during the whole life span of the mice, we analyzed the knee joints from 3-day-old and 5-, 12-, and 24-month-old animals (Figure S1A, B, C, D, and E), confirming the existence of CCN2 in the TG articular cartilage.

Since CCN2 enhances the expression of components of extracellular matrices, there was evidence of strong accumulation of type II collagen in the transgenic growth plate (Figure 1F). However, in the articular cartilage, only slightly, but not significantly enhanced accumulation of type II collagen was observed in the deep zone of the TG articular cartilage (Figure 1F and Figure S2A-1 and -2).

### CCN2 Overexpression Prevents Degenerative Changes in the Articular Cartilage in Aging Joints

**Figure 2:**
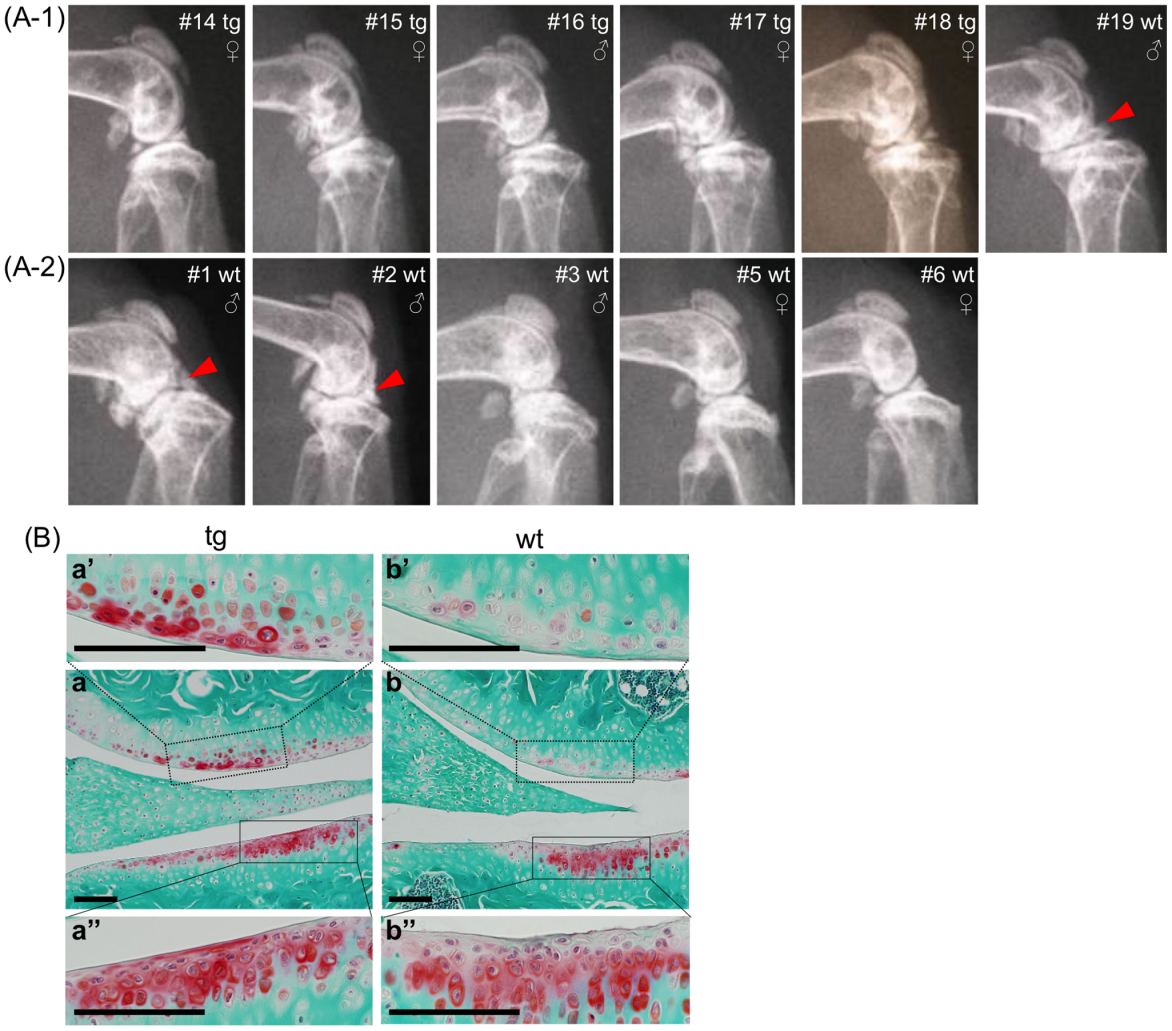
Morphological analysis of aged knee joints from CCN2 transgenic mice. (A) X-ray photographs of knee joints from littermates of 21- (A-1) and 18-month-old (A-2) mice. 50 percent (3 out of 6) of the knee joints from WT, but none of the TG mice, developed osteoarthritis-like phenotypes with a narrowing joint space and rough cartilage surfaces. Arrowheads indicate degenerative changes in the articular cartilage in WT joints. (B) Safranin-O-fast green staining of frontal sections of knee joints from 21-month-old TG and WT mice. a and b: whole view of joints at low magnification. Higher magnification of load-bearing regions of femur (a’ and b’) and tibia (a” and b”), which regions are indicated by the dotted boxes in a and b. Bars: 100 µm. More Safranin-O-positive chondrocytes remained on the surface of articular cartilage of TG mice than on that of the WT cartilage.

In order to assess possible protective effects of overexpressed CCN2 on the cartilage in aging joints, we analyzed knee joints of all littermates from 21-month-old (1 male TG, 1 male WT, and 4 females TG) and 18-month-old WT (3 WT males and 2 WT females) litters by X-ray analysis. In the WT knee joint, we observed osteoarthritis-like degenerative phenotypes, as indicated by a narrowing joint space and osteophyte-like protrusions (marked by the arrowhead), which were not seen in the transgenic joints. Fifty percent (3 out of 6) of the WT knee joints, but no TG joints, developed osteoarthritis-like changes (Figure 2A-1 and -2).

### CCN2 Overexpression Stabilizes Proteoglycans in Articular Cartilage

**Figure 3:**
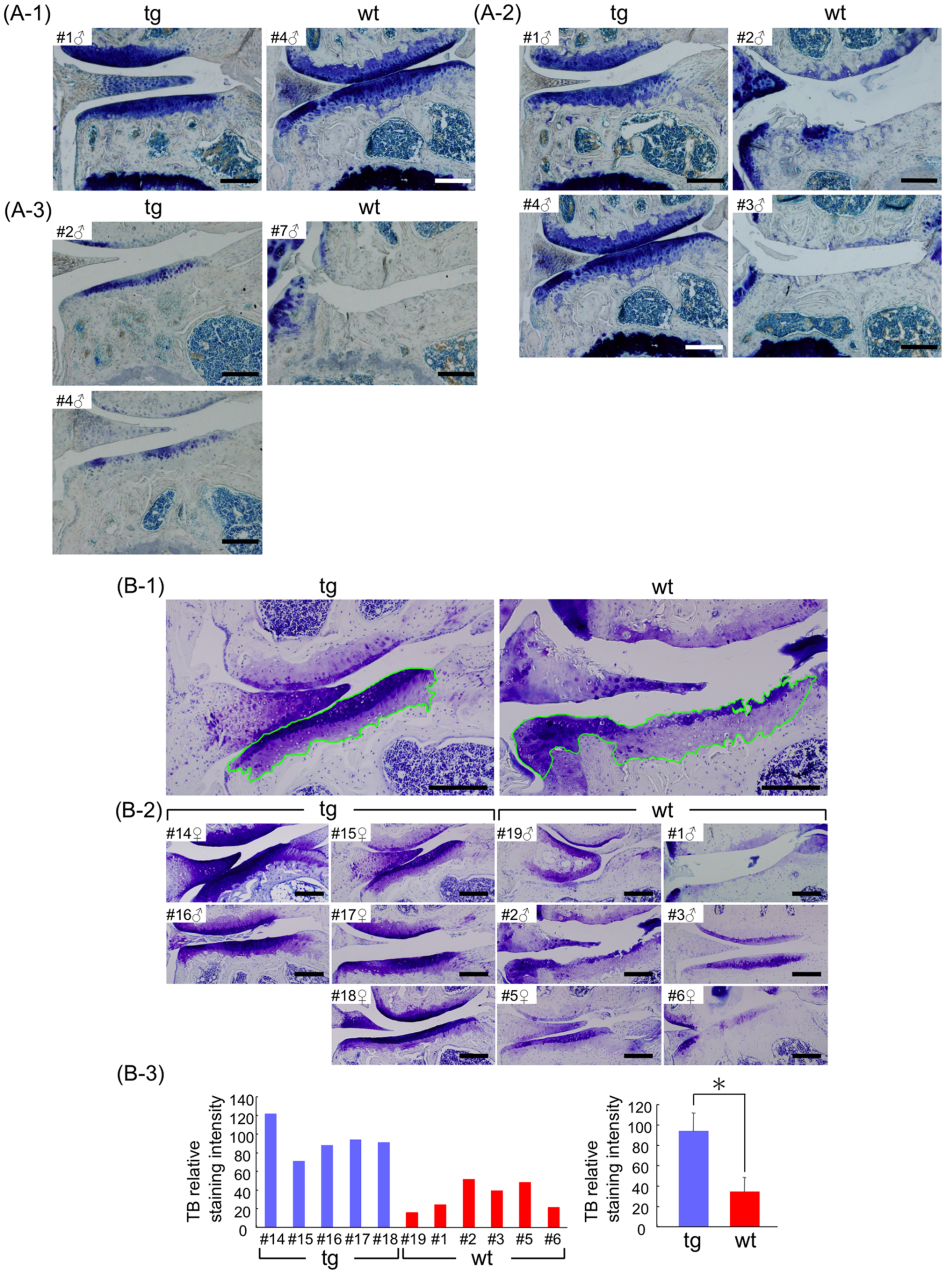
Enhanced proteoglycan accumulation in articular cartilage of CCN2 transgenic mice. (A) Toluidine blue (TB) staining of knee joints from 5- (A-1), 12- (A-2), and 24-month-old (A-3) male littermates [identify animal number in each age group] (Left side: TG, right side: WT). Bars: 200 µm. left: TG and right: WT littermates. The content of TB metachromasia-positive proteoglycans decreased with aging; however, TG articular cartilage showed a greater accumulation of proteoglycans than did the cartilage of WT littermates at all time points. (B) Histomorphometric analysis of TB staining of knee joints from 21-month-old littermates [male (TG, #16; WT, #19), female (all TG, #14, #15, #17, #18 )], and 18-month-old littermates [all WT (male, #1, #2, #3; female, #5, #6 )]. (B-1) Representative views of toluidine-blue stained medial tibial cartilage in the load-bearing region (measured area outlined in green). (B-2) TB-stained knee joints. Numbers indicate individual animals. Bars: 200 µm. (B-3) Results of densitometric analysis of specimens in B-2. Left: staining intensity from individual medial tibial cartilages. Right: mean value (Axiovision, Student’s t-test). TG cartilage showed a significantly higher amount of proteoglycan than the WT cartilage. *: *p*<0.0005.

Safranin-O staining in the superficial and middle layers of the articular cartilage was significantly enhanced in intensity in 21-month-old TG animals as compared with that for WT littermates (Figure 2B). Similarly, toluidine blue staining revealed a considerable loss of proteoglycans in the articular cartilage of the knee joints from 5-month- (Figure 3A-1), 12-month- (Figure 3A-2), and 24-month-old (Figure 3A-3) WT littermates compared with the TG animals. Statistical analysis of toluidine blue staining intensity of 21- and 18-month-old littermates showed enhanced proteoglycan accumulation in the TG articular cartilage compared with the accumulation in the WT animals (Figure 3B-1, -2, and -3). These findings indicate that after overexpression of CCN2, significantly more intact proteoglycans remained in the transgenic cartilage of aging animals than in the WT cartilage.

### CCN2 Suppresses Hypertrophic Differentiation of Deep-zone Articular Chondrocytes

**Figure 4:**
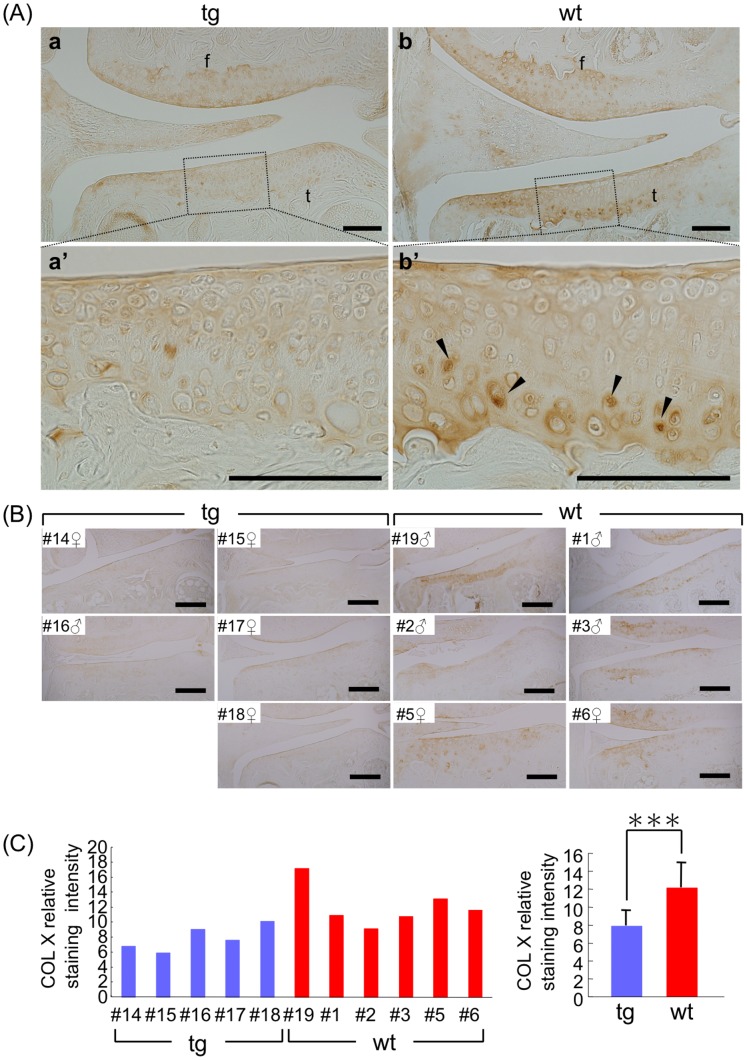
Suppression of age-related hypertrophic differentiation of deep-zone articular chondrocytes by overexpression of CCN2. Immunohistochemical staining of type X collagen in frontal sections of knee joint. (A) Typical staining of type X collagen of the TG (left) and WT (right) joints from 21-month-old mice. Bars: 100 µm. a and b: type X collagen staining of the medial portion of knee joint in the load-bearing region. Femora (f) and tibia (t) are presented. a’ and b’: higher magnification of load-bearing region indicated by the dotted box in a and b, respectively. Type X collagen-positive cells (arrowheads in b’) were observed in the deep zone of WT tibial articular cartilage, but not in those of TG cartilage. (B) Type X collagen staining of articular cartilage (knee joints) from 21- and 18-month-old littermates. Bars: 200 µm. (C) Results of densitometric analysis of type X collagen staining of tibial articular cartilage. Left: staining intensity of individual medial cartilage samples of tibia. Right: mean value. TG cartilage showed a significantly lower accumulation of type X collagen compared with the WT cartilage. ***: *p*<0.01.

Type X collagen (COL10) is expressed to some extent in the deep zone of normal adult human articular cartilage, but is enhanced during osteoarthritis-like progression [22]. Furthermore, it has been also found in the articulating surface zone of dog cartilage after stress-induced damage to cartilage in running dogs [39]. In 21- and 18-month-old WT mice, significant COL10 expression was detected in the deep calcified zone below the tidemark, whereas there was much less COL10 in the deep zone of the TG articular cartilage (Figure 4A). Statistical analysis of the staining intensity of articular cartilage from 21- and 18-month-old TG littermates showed significantly less staining compared with that for the WT (Figure 4B and C), indicating that overexpression of CCN2 may have suppressed the hypertrophic differentiation of the articular chondrocytes. This observation is in line with our previous report showing that CCN2 suppresses type X collagen expression in articular chondrocytes *in vitro* [6].

Type I collagen was also examined, as an indicator of fibrotic degeneration of cartilage [40], [41], and was detected in the WT articular cartilage both intracellularly and in the extracellular cartilage matrix; whereas it was restricted to the bone and meniscus in the TG joints (Figure S2B). These observations indicate the beginning of chondrocyte dedifferentiation in 21- and 18-month-old WT, but not in TG, articular cartilage.

### CCN2 Overexpression Suppresses Expression of MMP-13 in Superficial and Middle-zone Chondrocytes

**Figure 5:**
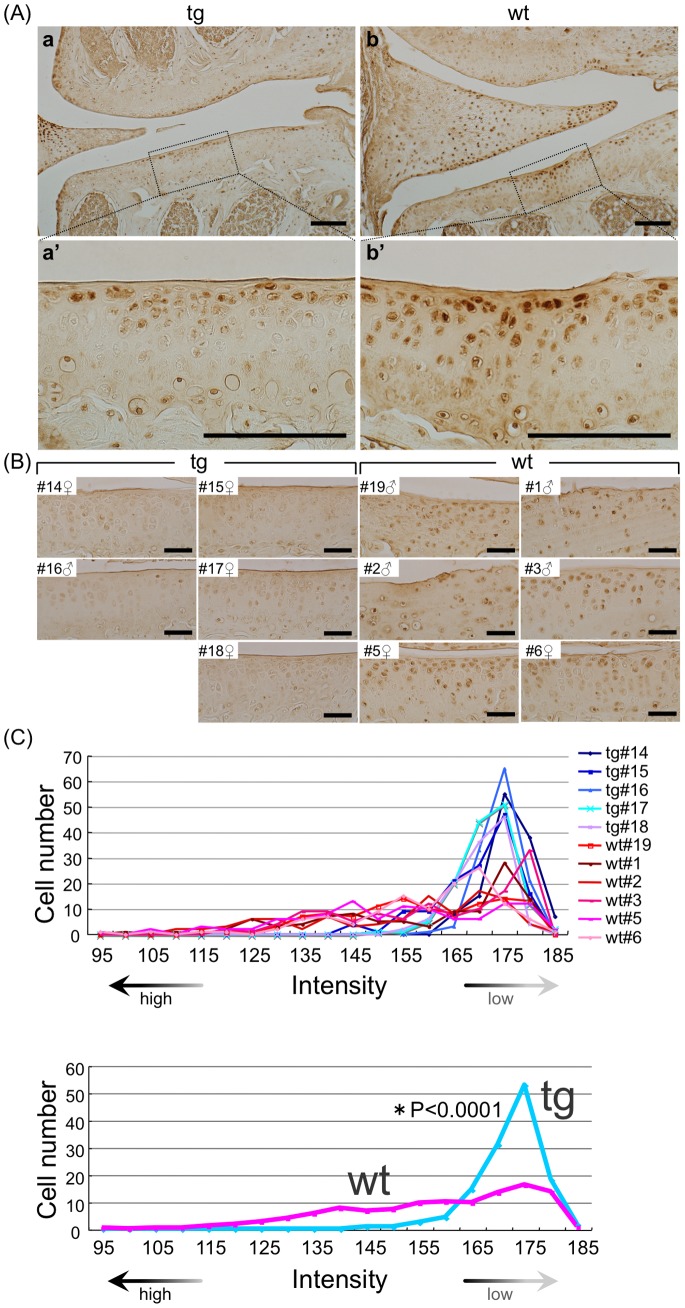
Suppression of MMP-13 expression in aging superficial and middle-zone articular chondrocytes by CCN2 overexpression. Immunohistochemical staining of MMP-13 in frontal sections of knee joint. (A) Typical staining of MMP13 of TG (left) and WT (right) knee joints of 21-month-old mice. Bars: 100 µm. a and b: MMP-13 staining of articular cartilage, and a’ and b’: higher magnification of medial tibial plateau in the load-bearing region indicated by the dotted box in a and b. In WT tibial articular cartilage, MMP-13-positive cells were seen in the surface zone. (B) MMP-13 staining of knee joints from 5 transgenic and 6 WT 21- and 18-month-old littermates (Left side: TG, right side: WT). Densitometric intensity of the cells in tibial articular cartilage from each animal was measured. Bars: 50 µm. (C) Histogram of MMP13-positive cells in medial cartilage of tibia. Upper: staining density of 5 transgenic and 6 WT samples. Lower: mean value for TG and WT. Distribution of staining intensity was analyzed by a computer software (Axiovision, chi-square test) that originally measures “brightness” (the reverse of staining intensity). Most TG chondrocytes were in the peak of low staining intensity, indicating low activity of MMP-13; whereas more WT chondrocytes were in the area of higher staining intensity, indicating higher MMP13 accumulation.

MMP-13, a protease expressed primarily in hypertrophic chondrocytes and involved in cartilage remodeling, was strongly expressed in superficial- and middle-zone chondrocytes above the tidemark in specimens from 21- and 18-month-old WT animals. In contrast, significantly fewer MMP-13-positive chondrocytes were seen in the TG articular cartilage (Figure 5A). Densitometric analysis of individual cells clearly showed a reduced distribution of the cells having high staining intensity in TG articular cartilage (Figure 5B and C). These findings confirm that WT articular chondrocytes underwent hypertrophic differentiation and expressed MMP-13 with aging, and indicate that CCN2 expression suppressed this differentiation associated with MMP-13 expression.

### CCN2 Overexpression Enhances Cell Proliferation in Articular Cartilage of Aged Mice

**Figure 6:**
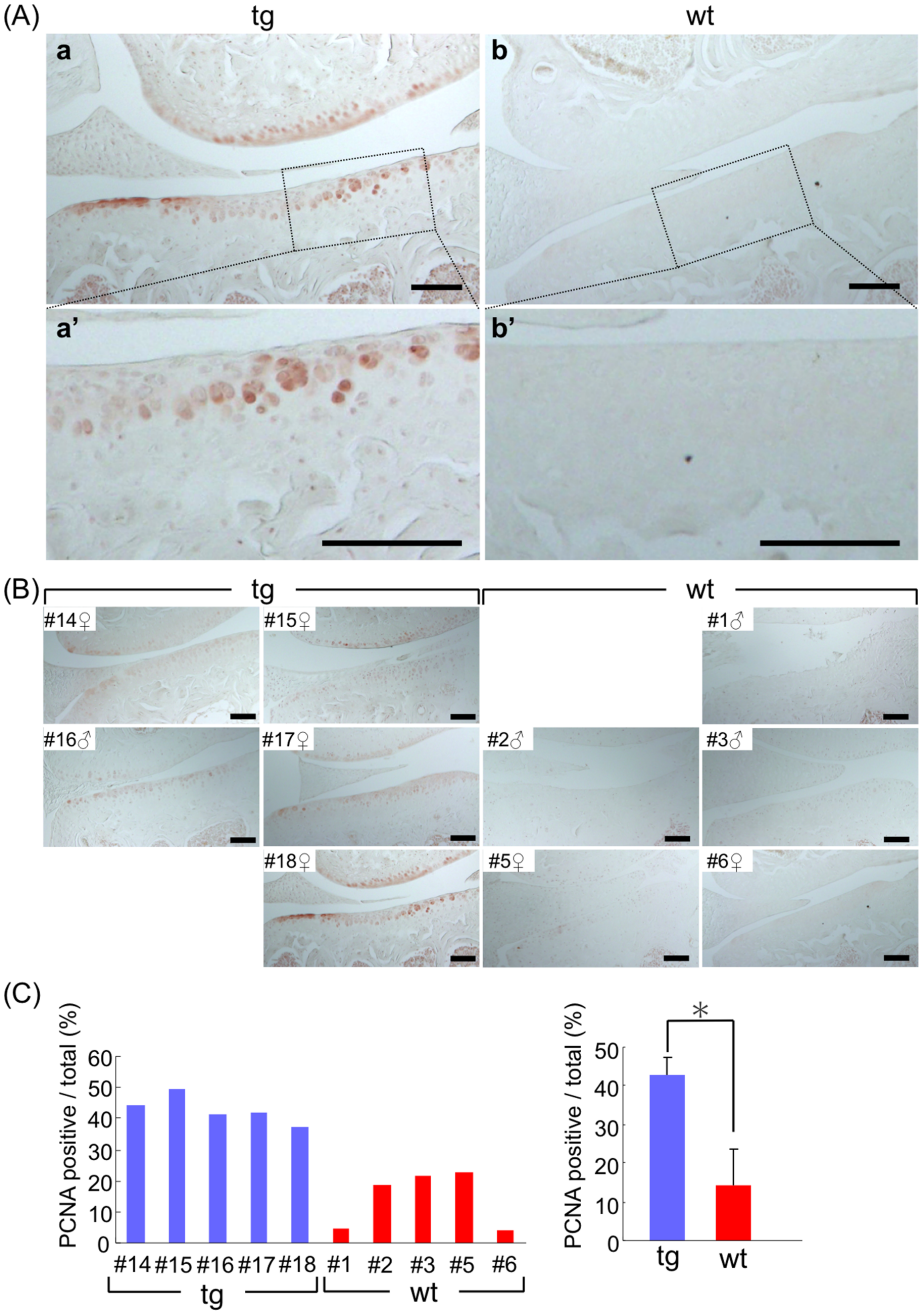
Sustaining proliferation of articular chondrocytes in aged mice by CCN2 overexpression. (A) PCNA staining in chondrocytes of knee joint cartilage (tibial plateau, frontal sections) of TG joints from 21-month-old mice (a) and of WT joints (b) from 18-month-old mice. a’ and b’: higher magnification of the medial tibial plateau in the load-bearing region indicated by the dotted box in a and b. Bars: 100 µm. In TG articular cartilage, PCNA-positive chondrocytes were observed in the surface area. Bars: 100 µm. (B) PCNA staining of knee joints from 21-month-old littermates and 18-month-old littermates. (C) Left: Percentage of PCNA-positive cells per total cells in TG or WT tibia medial cartilage of individual mice. Right: mean value for TG and WT. TG cartilage showed a significantly higher ratio of PCNA-positive chondrocytes than WT cartilage. *: *p*<0.001.

In TG articular cartilage, a significantly larger number of PCNA-positive chondrocytes were detected in superficial layer compared with their number in the WT cartilage (Figure 6A). Their location corresponded to the location of CCN2-positive cells, indicating that CCN2 may have accelerated the proliferation of articular chondrocytes. By contrast, much less cell proliferation was seen in the WT articular cartilage (Figure 6B and C).

### CCN2 Prevents Age-related Degeneration of Proteoglycans in Articular Cartilage

**Figure 7:**
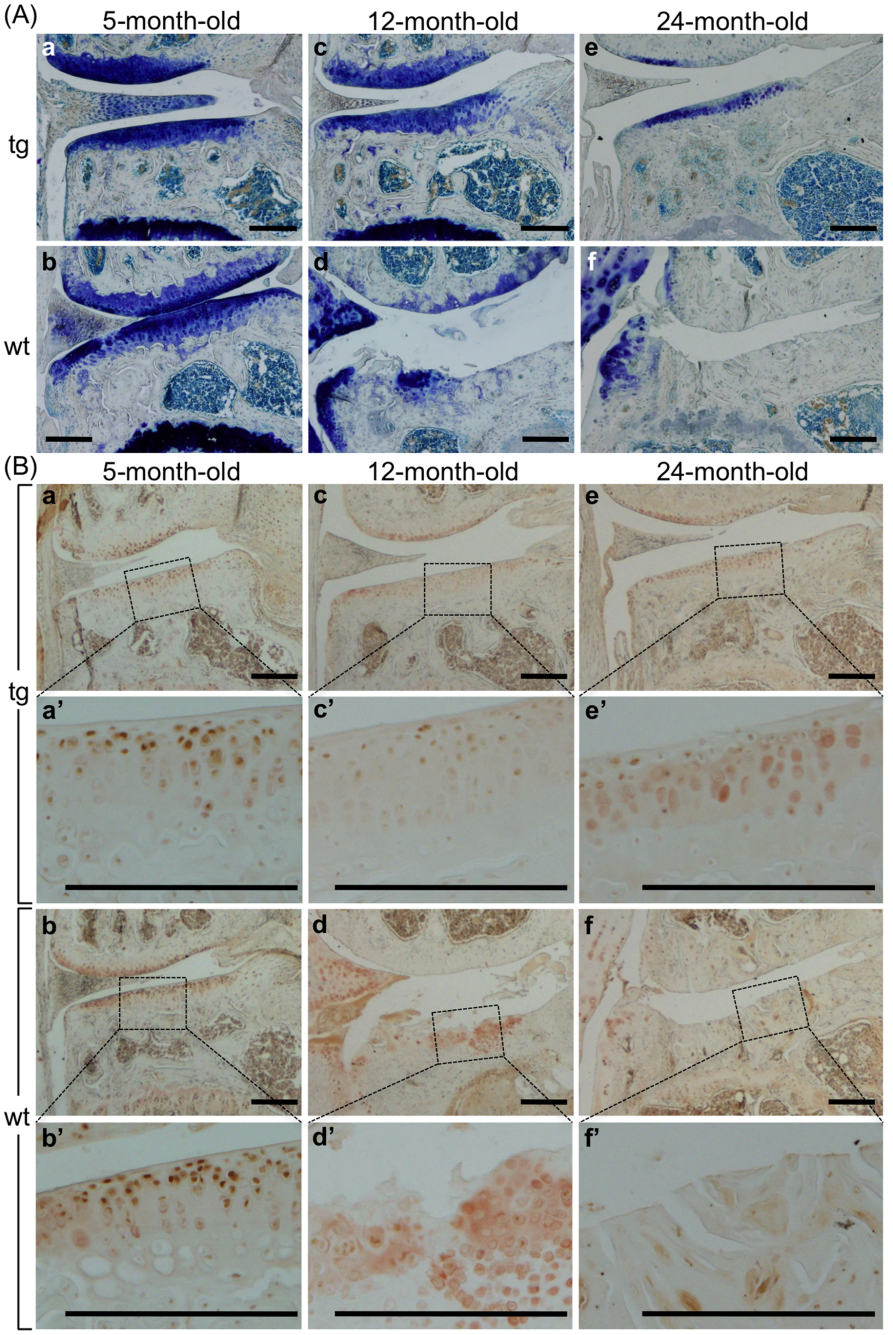
Preventing age-related degeneration of proteoglycans in articular cartilage by CCN2 overexpression. (A) Toluidine blue staining of frontal sections of medial portion of TG (a, c, e) and WT (b, d, f) littermate knee joints from 5- (a and b), 12- (c and d), and 24- (e and f) month-old male mice. TB staining indicated that WT articular cartilage was degraded with age, and finally the whole layer was lost after 24 months. (B) Immunohistochemical staining of aggrecan neoepitope in frontal sections of the medial portion of TG (a, c, e) and WT (b, d, f) littermate knee joints from 5- (a and b), 12- (c and d), and 24-month-old (e and f) mice. a’, b’, c’, d’, e’, and f’: higher magnification of the medial tibial plateau in the load-bearing region, indicated by the dotted box in a, b, c, d, e, and f, respectively. Bars: 200 µm. In the WT tibia (d and d’) even the remaining matrices were slightly degraded, as shown by the aggrecan neoepitope staining. TG cartilage showed a minor decrease in staining for the aggrecan neoepitope (a’, c’, and e’), but not severe as in the WT cartilage (b’, d’, and f’).

Toluidine blue staining showed dramatically decreased proteoglycan accumulation in WT articular cartilage after aging, but less changes in TG animals (Figure 7A). To estimate the degenerative changes in WT articular cartilage, we detected the aggrecan neoepitope. In the WT tibial articular cartilage, the level of the aggrecan neoepitope increased dramatically in 12-month-old mice, whereas it was reduced in the TG cartilage (Figure 7B). In specimens obtained from 24-month-old animals, no staining was seen in WT knee joints, due to the loss of articular cartilage; whereas in the TG articular cartilage, aggrecan neoepitope staining was at the level seen in the 5-month-old mice (Figure 7B). 5-, 12-, and 24-month-old littermates (all male) were also analyzed (Figure S3A, B, and C). WT, but not TG, mice at 12 months of age developed severe degenerative changes in their knee joints.

### WT Chondrocytes Respond to Cyclic Tension Stress (CTS) by Enhanced Expression of Catabolic and Anabolic Cartilage Genes, whereas CCN2-overexpressing Chondrocytes Respond by Suppression of these Genes

**Figure 8:**
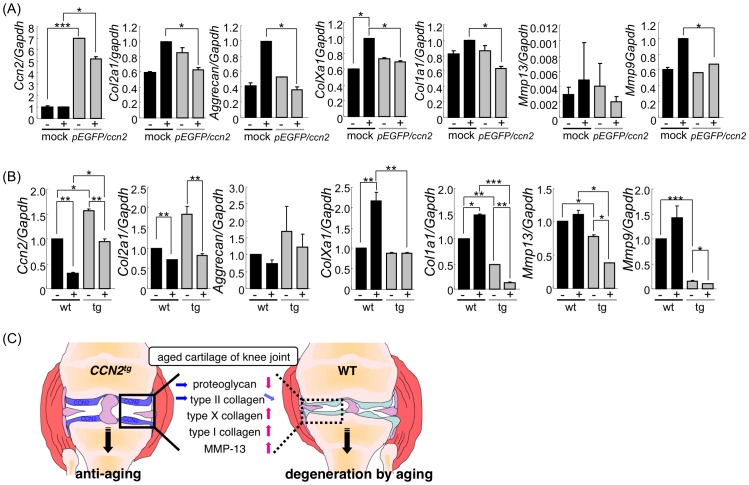
CCN2 overexpression reduces cyclic tension stress (CTS)-induced gene expression changes in cultured chondrocytes. (A) Gene expression analysis of cartilage matrix constituents and proteases of GFP-CCN2 overexpressing rib chondrocytes or mock transfectants after application of cyclic tension stress (CTS) (+). Control cells without CTS (−). Primary cultures of mouse rib chondrocytes overexpressing GFP-CCN2 or GFP as a control were subjected to CTS at 0.5 Hz, with 6% elongation, for 12 hours; and gene expression was then measured by RT-PCR. The expression of transfected *Ccn2* was decreased after CTS. The expression of *Col2a1, Aggrecan, ColXa1, Col1a1, Mmp13*, and *Mmp9* genes was stimulated after CTS application to mock cells, but was slightly reduced or remained equal when CTS was applied to CCN2-overexpressing rib chondrocytes after CTS. *: *p*<0.05 **: *p*<0.01 ***: *p*<0.005 (B) Gene expression analysis of cartilage matrix constituents and proteases in cultures of epiphyseal chondrocytes from 6-day-old TG and WT mice, subjected to after CTS as in (A). Contrary to that in rib chondrocytes, the expression of *Col2a1* and *Aggrecan* was decreased when CTS was applied to WT cells. The expression of *Col1a1, ColXa1,* and *Mmp9* genes was stimulated by CTS in WT cells, but down-regulated in the TG chondrocytes. *Mmp13* gene was also reduced in the TG chondrocytes by CTS. *: *p*<0.05 **: *p*<0.01 ***: *p*<0.005 (C) Cartilage-specific overexpression of CCN2 under the control of type II collagen promoter alleviated the development of degenerative changes in aging articular cartilage such as decreases in proteoglycan and type II collagen and enhanced expression of type X and I collagens and MMP-13.

There is ample evidence that mechanical stress applied to chondrocytes activates the expression of genes involved in chondrocyte degeneration and cartilage destruction, such as those of MMP-13 [42] or ADAMTS-5 [43]. In order to determine whether the CCN2 also impaired the expression of stress–induced genes in chondrocytes, we exposed CCN2-overexpressing and mock-transfected rib chondrocytes to CTS, and then examined them for changes in gene expression by using real-time PCR (Figure 8A). Overexpression of the *Ccn2* in the transfected cells was maintained during the culture period (Figure S3D, E, and F). Application of CTS decreased the expression level of *Ccn2* in *Ccn2*-transfected cells; however this level was still elevated as compared with that in the mock-transfectants (Figure 8A). Interestingly, mock-transfected rib chondrocytes responded to cyclic stress by enhanced expression of *Col2a1, Aggrecan, Col10a1, Col1a1*, and *Mmp9* mRNA; whereas CCN2-overexpressing cells responded by undergoing slightly reduced expression of these genes, except for *Mmp13* (Figure 8A). A similar response was observed in cultures of TG and WT epiphyseal chondrocytes (Figure 8B). Similar to the case of CCN2-transfected rib chondrocytes, CCN2 overexpression caused a significant increase in *Col2a1* and *Aggrecan* expression, and CTS caused a reduction in *Col2a1* and *Aggrecan* expression in CCN2 transgenic epiphyseal chondrocytes. WT epiphyseal chondrocytes, however, did not respond to CTS by enhancing their expression of *Col2a1* and *Aggrecan.* Similar to that in rib chondrocytes, *Col10a1* expression was only stimulated by CTS in WT epiphyseal chondrocytes cells, but was not affected in CCN2-overexpressing cells.

Also with respect to expression of *Mmps*, rib and epiphyseal chondrocytes responded to CTS in a similar manner: Expression of *Mmp13* was stimulated by CTS in mock-transfected and WT chondrocytes, but impaired in CCN2-overexpressing rib and epiphyseal chondrocytes. Expression of *Mmp9* was also stimulated in mock-transfected and WT cells, but not altered in CCN2-overexpressing cells (Figure 8A, B, and Supporting Information S1).

These results indicate that overexpression of *Ccn2* stimulated the expression of cartilage matrix genes in hyaline chondrocytes, but conferred resistance to degenerative changes generated by mechanical stress.

## Discussion

In this study, we evaluated the *in vivo* effect of cartilage-specific overexpression of CCN2 on the maintenance of knee joint cartilage in aging CCN2-transgenic mice. In the knee joints of 12-, 18-, 21-, and 24-month-old WT mice, articular cartilage showed degenerative changes such as proteoglycan loss, surface erosion, enhanced chondrocyte hypertrophy, and enhanced type I collagen expression, all of which are characteristic changes in aging mice [44], [45] and similar to the age-related changes seen in STR/Ort mice [31]. CCN2 overexpression in the articular cartilage under the control of the *Col2a1* promoter alleviated these degenerative changes and signs of chondrocyte degeneration 1) by promoting *Aggrecan* and *Col2a1* synthesis, 2) by suppressing synthesis and deposition of markers of chondrocyte hypertrophy (*Col10a1*), chondrocyte dedifferentiation (*Col1a1*), and cartilage degradation (*Mmp9* and *Mmp13*), and 3) by promoting chondrocyte proliferation. These results indicate that the overexpression of CCN2 conferred a protective effect on articular cartilage against degenerative changes with aging by promoting chondrocyte proliferation and proteoglycan synthesis.

In TG mice, overexpressed CCN2 protein accumulated in the superficial and middle zones of the articular cartilage, and the chondrocytes remained proliferative in these layers. Safranin-O and toluidine blue staining indicated significantly more intact proteoglycans remaining in 5-, 12-, 21-, and 24-month-old TG articular cartilage than in WT cartilage. Similar, slightly enhanced deposition of type II collagen was found in CCN2 TG articular cartilage as compared with that in WT littermates. These findings are consistent with several reports showing stimulation of *Col2a1* and *Aggrecan* expression in cartilage by CCN2 [3], [5].

Under physiological conditions, only chondrocytes of the deep zone below the tidemark, but not those of the superficial and middle zone of young articular cartilage undergo hypertrophic differentiation; whereas those in the superficial and middle zone maintain a stable phenotype as a permanent cartilage [22], [23]. With aging or after exposure to an excessive mechanical load, however, partial hypertrophy of chondrocytes in the superficial and middle zones associated with enhanced type X collagen expression has been reported to occur in mouse and canine articular cartilage [46], [47], [48]. In OA, pathologic expression of type X collagen and that of other markers of hypertrophy such as annexin VI, alkaline phosphatase, osteopontin, and osteocalcin have been observed in deep, superficial and middle zones of articular cartilage [22], [47], [49].

Type X collagen deposition in aging articular cartilage was suppressed by CCN2 overexpression. The real-time PCR analysis of CCN2-overexpressing rib and epiphyseal chondrocytes after the addition of CTS did not indicate suppression of *Col10a1* expression at the mRNA level; however, since CTS stimulated the expression of *Col10a1* and *Col1a1* in both mock-transfected rib chondrocytes and WT epiphyseal chondrocytes. The reduced type X and type I collagen deposition in CCN2 transgenic cartilage is consistent with the *Col2a1*- and *Aggrecan*-stimulating effect of CCN2, which stabilizes the hyaline phenotype of chondrocytes. The stimulation of chondrocyte proliferation by CCN2 might have additionally contributed to stabilization of the cartilage phenotype in CCN2 transgenic mice.

Numerous studies have shown that moderate mechanical stress stimulates matrix synthesis of articular cartilage, whereas excess mechanical stress is a major cause of secondary osteoarthritis [20]. Adaptation of articular cartilage to mechanical stimulation is related to the interaction between the chondrocytes and the matrix [50], [51]. Accumulated extracellular matrices may act as a shock absorber to the mechanical load in the joints. On the other hand, a recent report showed that the postnatal ablation of Sox9 dramatically decreases type II collagen mRNA and aggrecan contents; however, no histopathological signs of osteoarthritis were observed [52], indicating that the amount of extracellular matrix in articular cartilage is important but may not be necessary for the development of degradative changes in articular cartilage.

MMP-13, a protease produced by late hypertrophic chondrocytes [18] and which potently degrades cartilage matrix with a preference for type II collagen, is known to be induced in OA articular cartilage and to be functionally involved in OA pathogenesis [53]. This proteinase has been suggested to be induced in response to proinflammatory cytokines such as tumor necrosis factor α (TNFα), interleukin-1 (IL-1), and IL-6 in articular cartilage under pathologic conditions, such as found in OA and rheumatoid arthritis (RA) [54]. However, it is questionable whether these cytokines play significant roles in the development of OA.

*Col2a1* and *Aggrecan* expression was stimulated in mock rib chondrocytes by CTS, but slightly inhibited or not affected in WT epiphyseal chondrocytes (Figure 6B and C). These results may relate to the difference in the source of chondrocytes and their differential response to CTS [55], [56], [57].

Recent reports claimed that applying excessive load to a joint induces neovascularization at the region of the articular cartilage from synovium or tendon; subsequently, endochondral ossification can be initiated and osteophytes are formed [21]. Although CCN2 also promotes angiogenesis [3], [9] the CCN2-overexpressing area in the articular cartilage was spatially separated from the peripheral surface region. In addition, cartilage contains many anti-angiogenic factors such as chondromodulin-1 [58] and TIMP-2 [59]. Altogether, the protective effects of CCN2 in articular cartilage against aging and degeneration such as by stimulating chondrocyte proliferation and proteoglycan synthesis, as well as by suppressing protease expression seem to outweigh any potential angiogenic effects of CCN2 in cartilage. This is in line with our previous reports showing that CCN2 stimulates the proliferation and differentiation, but not hypertrophy, of articular chondrocytes [6].

In conclusion, we have demonstrated that overexpression and accumulation of CCN2 in the extracellular matrices protects articular cartilage from age-related degenerative changes. Adult transgenic articular cartilage showed higher levels of proteoglycans as compared with those of littermates. *Ccn2* transgene expression levels were also enhanced in transgenic cartilage, as expected; although their expression levels were lower than those of growing and younger ages. In a recent study we showed that *Ccn2* transgenic mice have a strongly enhanced accumulation of extracellular matrix, accelerated endochondral ossification, and extended bone growth [5]. The protective effects of CCN2 against degenerative changes in CCN2-overexpressing TG chondrocytes may have also been due to the enhanced accumulation of cartilaginous extracellular matrices at younger ages. Enhanced matrix accumulation may also explain the lower responsiveness of CCN2 transgenic chondrocytes to excess mechanical stress-induced changes (Figure 8C). The cellular mechanism of the cartilage-protective effects of CCN2 still needs to be elucidated.

## Supporting Information

Figure S1 **Accumulation of CCN2 in transgenic articular cartilage at different age and comparison of CCN2 accumulation in transgenic littermates.** (A–E) Immunohistochemical staining of CCN2. Knee joints from 3-day (A), 60-day (B), 5-month (C), 12- (D), and 24-month-old (E) littermates. (F-1) Comparison and densitometric analysis of medial knee joints from 21- and 18-month-old littermates [identify animal number in each age group] (Left side: TG, right side: WT). Staining area was circled, and the staining intensity was measured. Bars: 200 µm. (F-2) Left: staining intensity of individual medial tibial cartilages. Right: mean value. TG cartilage showed a significantly higher amount of accumulated CCN2 compared with the WT cartilage. *: *p*<0.005.
(TIF)

Figure S2 **Comparison of type II and I collagen accumulation in articular cartilage between CCN2 transgenic littermates.** (A) Immunohistochemical staining of type II collagen. (A-1) Comparison and densitometric analysis of medial knee joints from 21- and 18-month-old littermates [identify animal number in each age group] (Left side: TG, right side: WT). Bars: 200 µm. (A-2) The staining area of each specimen was circled; and the staining intensity of it was measured. Left: staining intensity from individual medial tibial cartilages. Right: mean value. Accumulation of type II collagen in the medial tibial cartilage was not significantly different between TG and WT. *p* = 0.496 (B) Immunohistochemical staining of type I collagen in frontal sections of knee joints. (B-1) Typical staining of type I collagen of TG (left) and WT (right) joints from 21-month-old mice. Bars: 100 µm. Upper photos: type I collagen staining of the medial side of knee joints in the load-bearing region. Lower photos: higher magnification of load-bearing region indicated by the dotted box in the upper photos. In WT tibial articular cartilage, type I collagen-positive cells are marked by arrowheads. (B-2) Type I collagen staining of knee joints from 21- and 18-month-old littermates [identify animal number in each age group] (Left side: TG, right side: WT). (B-3) The staining area was circled; and the staining intensity of it was measured. Left: staining intensity of individual medial cartilage of tibia. Right: mean value. TG cartilage showed a significantly lower amount of type I collagen deposition than WT cartilage. ***: *p*<0.01.
(TIF)

Figure S3 **Detection of aggrecan neoepitope in articular cartilage from transgenic littermates at different age and expression analysis of pEGFP/ccn2 vector in chondrocytes.** (A–C) Immunohistochemical staining of aggrecan neoepitope of medial knee joints from 5- (A), 12- (B), and 24-month-old (C) whole littermates [identify animal number in each age group]. Lower panels show magnified load-bearing regions of tibial articular cartilage indicated by the dotted boxes in the upper panels. In WT articular cartilage from 12-month-old mice, aggrecan neoepitope-positive extracellular matrices were observed in the surface area; and in other WT littermates at 12 months of age (B, lower panel) and at 24 months of age (C, lower panel), almost no staining occurred, due to the loss of articular cartilage. In contrast, TG articular cartilage, which was quite intact, showed no change in staining intensity (A, B, and C, upper panel). Bars: 200 µm. (D–F) Expression analysis of GFP-CCN2 in primary rib chondrocytes that had been transfected with GFP-CCN2 expression vector or empty vector (mock). (D) Immunoblot analysis of overexpressed GFP-CCN2 in chondrocytes, as performed with anti-GFP antibody. Primary rib chondrocytes were isolated and transfected with the GFP-CCN2 or mock expression vector. The cells were cultured in ex chambers as in the case of CTS-loaded cells. The cells were lysed by lysis buffer (10 mM Na-phosphate buffer at pH7.2, 150 mM NaCl, 1% Triton X-100, 0.1% SDS, 1 mM DTT, 0.1 mM PMSF), and the same amount of proteins was loaded into 12% SDS-PAGE gels. (E) Gene expression analysis of overexpressed *Ccn2* by real-time PCR. The cells, which were prepared by the same methods as indicated in (D) were used for preparation of total RNA. For real-time PCR analysis, primer sets were designed for the outside of the CCN2-coding region, and standardization was done with *Gapdh*. (F) Fluorescent images of GFP-CCN2-overexpressing cells. Before the cells had reached confluence, they fixed and stained with DAPI. a: GFP, b: DAPI, and c: merge.
(TIF)

Supporting Information S1 **Gene expression analysis of cartilage matrix constituents and proteases of epiphyseal chondrocytes from 6-day-old TG and WT mice after CTS.** The experiment was done several times, and the most typical results were shown in Figure 8.
(TIF)
