# Supplementary figures and images for: CCN Family Member 2/Connective Tissue Growth Factor (CCN2/CTGF) Has Anti-Aging Effects That Protect Articular Cartilage from Age-Related Degenerative Changes
Source: PLoS One. 2013 Aug 12;8(8):e71156. doi: 10.1371/journal.pone.0071156 (PMC3741357; doi:10.1371/journal.pone.0071156)

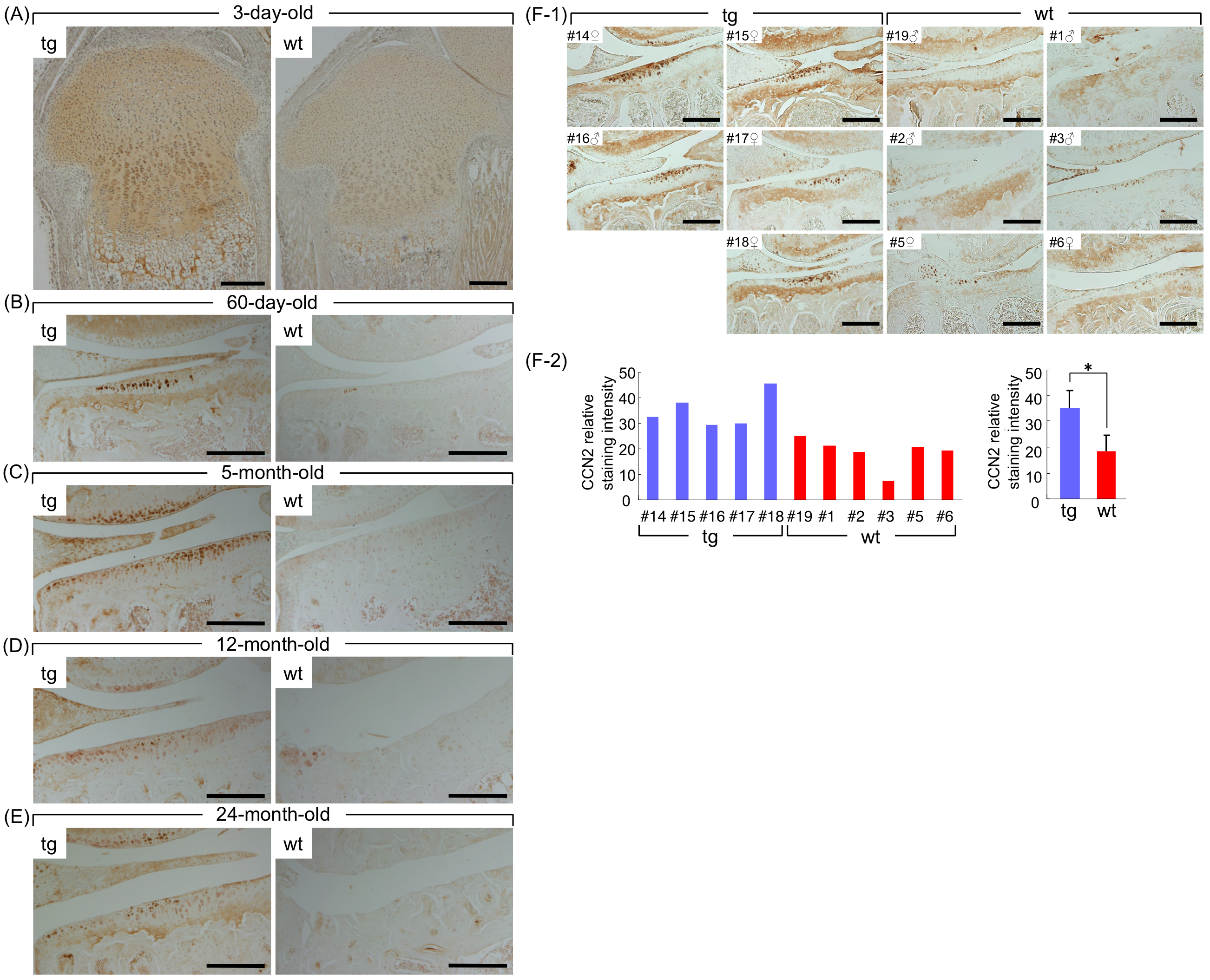

Supplement: Figure S1 — Accumulation of CCN2 in transgenic articular cartilage at different age and comparison of CCN2 accumulation in transgenic littermates. (A–E) Immunohistochemical staining of CCN2. Knee joints from 3-day (A), 60-day (B), 5-month (C), 12- (D), and 24-month-old (E) littermates. (F-1) Comparison and densitometric analysis of medial knee joints from 21- and 18-month-old littermates [identify animal number in each age group] (Left side: TG, right side: WT). Staining area was circled, and the staining intensity was measured. Bars: 200 µm. (F-2) Left: staining intensity of individual medial tibial cartilages. Right: mean value. TG cartilage showed a significantly higher amount of accumulated CCN2 compared with the WT cartilage. *: p<0.005. (TIF) [file pone.0071156.s001.tif]

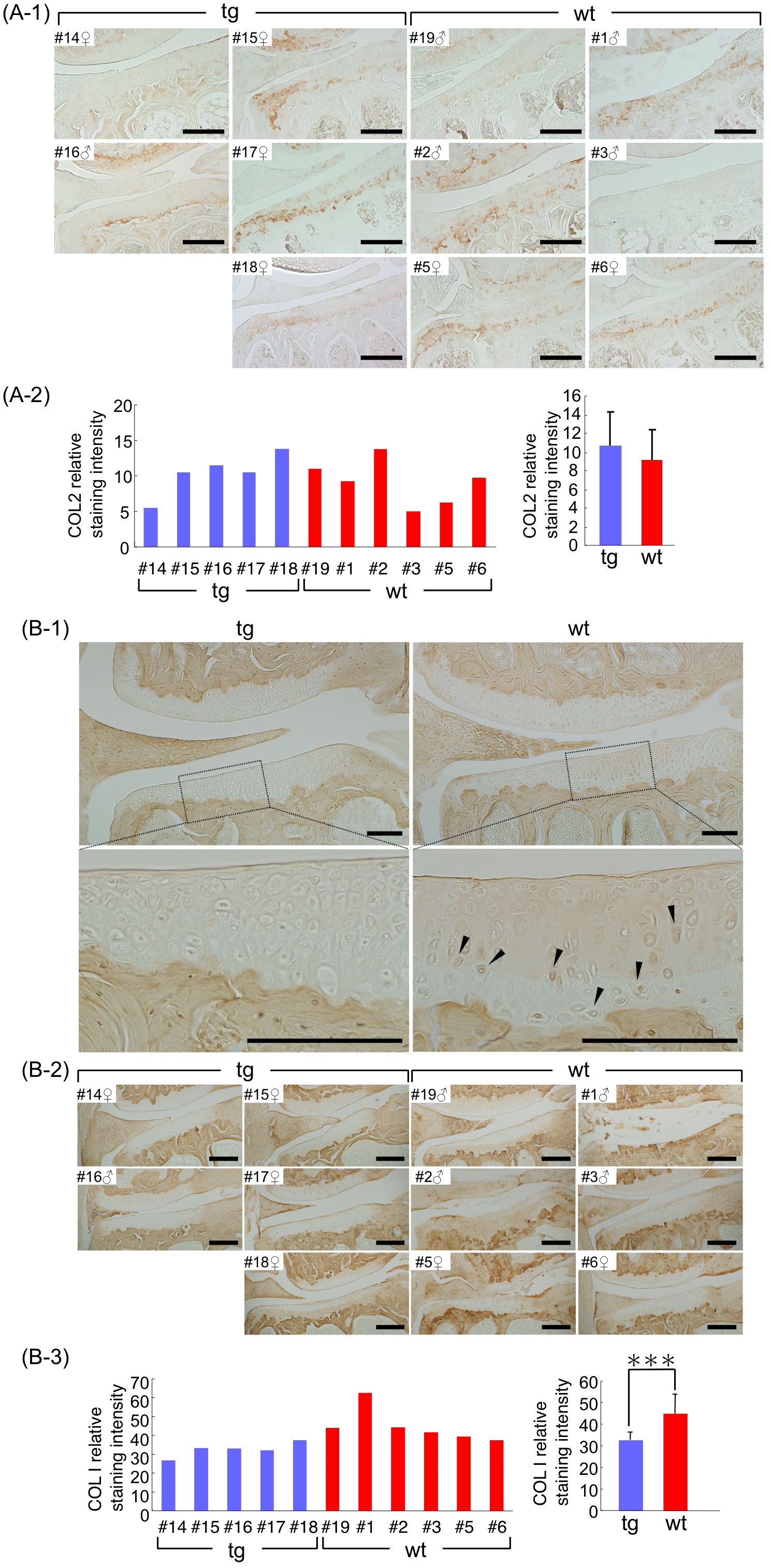

Supplement: Figure S2 — Comparison of type II and I collagen accumulation in articular cartilage between CCN2 transgenic littermates. (A) Immunohistochemical staining of type II collagen. (A-1) Comparison and densitometric analysis of medial knee joints from 21- and 18-month-old littermates [identify animal number in each age group] (Left side: TG, right side: WT). Bars: 200 µm. (A-2) The staining area of each specimen was circled; and the staining intensity of it was measured. Left: staining intensity from individual medial tibial cartilages. Right: mean value. Accumulation of type II collagen in the medial tibial cartilage was not significantly different between TG and WT. p = 0.496 (B) Immunohistochemical staining of type I collagen in frontal sections of knee joints. (B-1) Typical staining of type I collagen of TG (left) and WT (right) joints from 21-month-old mice. Bars: 100 µm. Upper photos: type I collagen staining of the medial side of knee joints in the load-bearing region. Lower photos: higher magnification of load-bearing region indicated by the dotted box in the upper photos. In WT tibial articular cartilage, type I collagen-positive cells are marked by arrowheads. (B-2) Type I collagen staining of knee joints from 21- and 18-month-old littermates [identify animal number in each age group] (Left side: TG, right side: WT). (B-3) The staining area was circled; and the staining intensity of it was measured. Left: staining intensity of individual medial cartilage of tibia. Right: mean value. TG cartilage showed a significantly lower amount of type I collagen deposition than WT cartilage. ***: p<0.01. (TIF) [file pone.0071156.s002.tif]

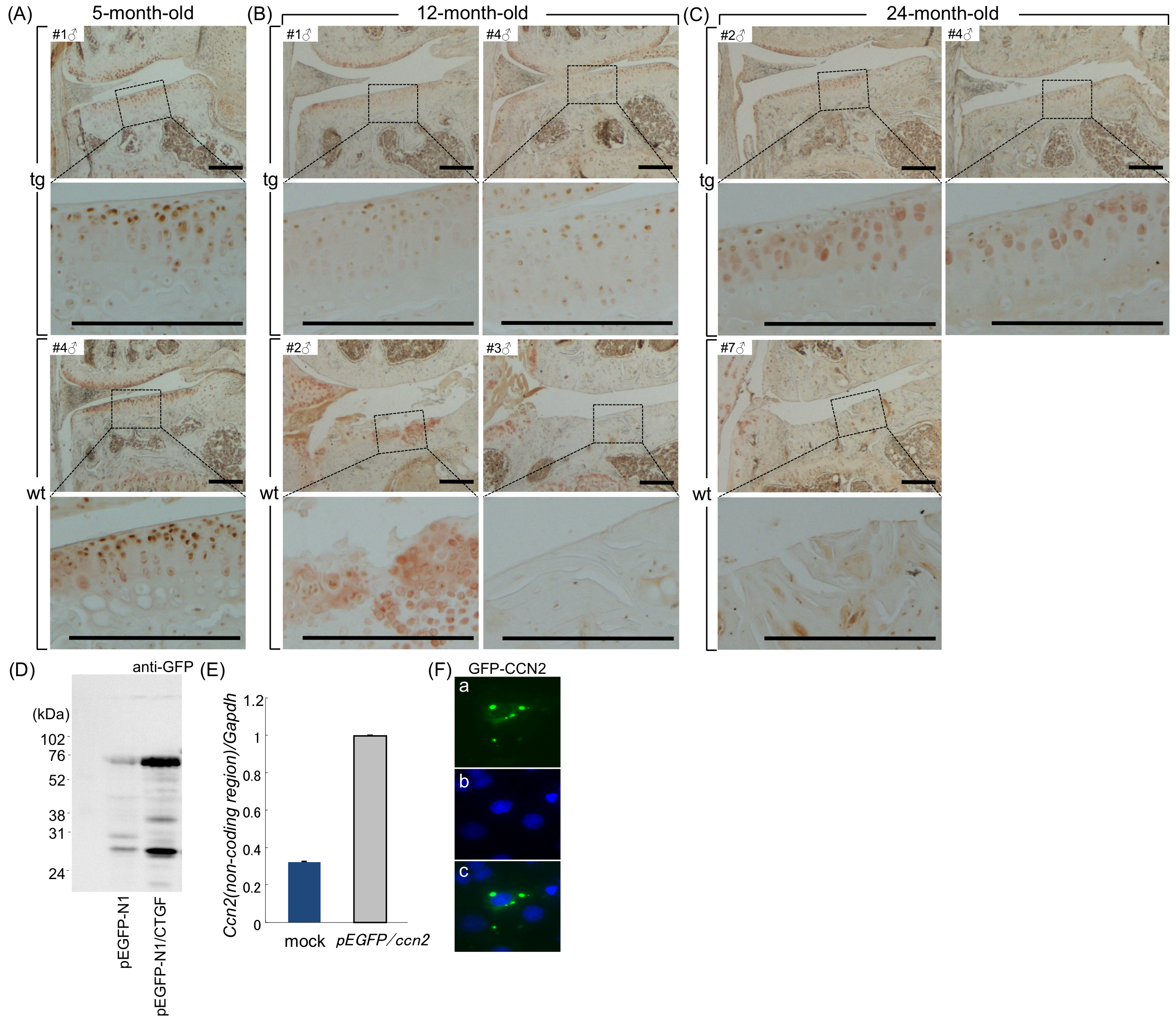

Supplement: Figure S3 — Detection of aggrecan neoepitope in articular cartilage from transgenic littermates at different age and expression analysis of pEGFP/ccn2 vector in chondrocytes. (A–C) Immunohistochemical staining of aggrecan neoepitope of medial knee joints from 5- (A), 12- (B), and 24-month-old (C) whole littermates [identify animal number in each age group]. Lower panels show magnified load-bearing regions of tibial articular cartilage indicated by the dotted boxes in the upper panels. In WT articular cartilage from 12-month-old mice, aggrecan neoepitope-positive extracellular matrices were observed in the surface area; and in other WT littermates at 12 months of age (B, lower panel) and at 24 months of age (C, lower panel), almost no staining occurred, due to the loss of articular cartilage. In contrast, TG articular cartilage, which was quite intact, showed no change in staining intensity (A, B, and C, upper panel). Bars: 200 µm. (D–F) Expression analysis of GFP-CCN2 in primary rib chondrocytes that had been transfected with GFP-CCN2 expression vector or empty vector (mock). (D) Immunoblot analysis of overexpressed GFP-CCN2 in chondrocytes, as performed with anti-GFP antibody. Primary rib chondrocytes were isolated and transfected with the GFP-CCN2 or mock expression vector. The cells were cultured in ex chambers as in the case of CTS-loaded cells. The cells were lysed by lysis buffer (10 mM Na-phosphate buffer at pH7.2, 150 mM NaCl, 1% Triton X-100, 0.1% SDS, 1 mM DTT, 0.1 mM PMSF), and the same amount of proteins was loaded into 12% SDS-PAGE gels. (E) Gene expression analysis of overexpressed Ccn2 by real-time PCR. The cells, which were prepared by the same methods as indicated in (D) were used for preparation of total RNA. For real-time PCR analysis, primer sets were designed for the outside of the CCN2-coding region, and standardization was done with Gapdh. (F) Fluorescent images of GFP-CCN2-overexpressing cells. Before the cells had reached confluence, they fixed and stai [file pone.0071156.s003.tif]

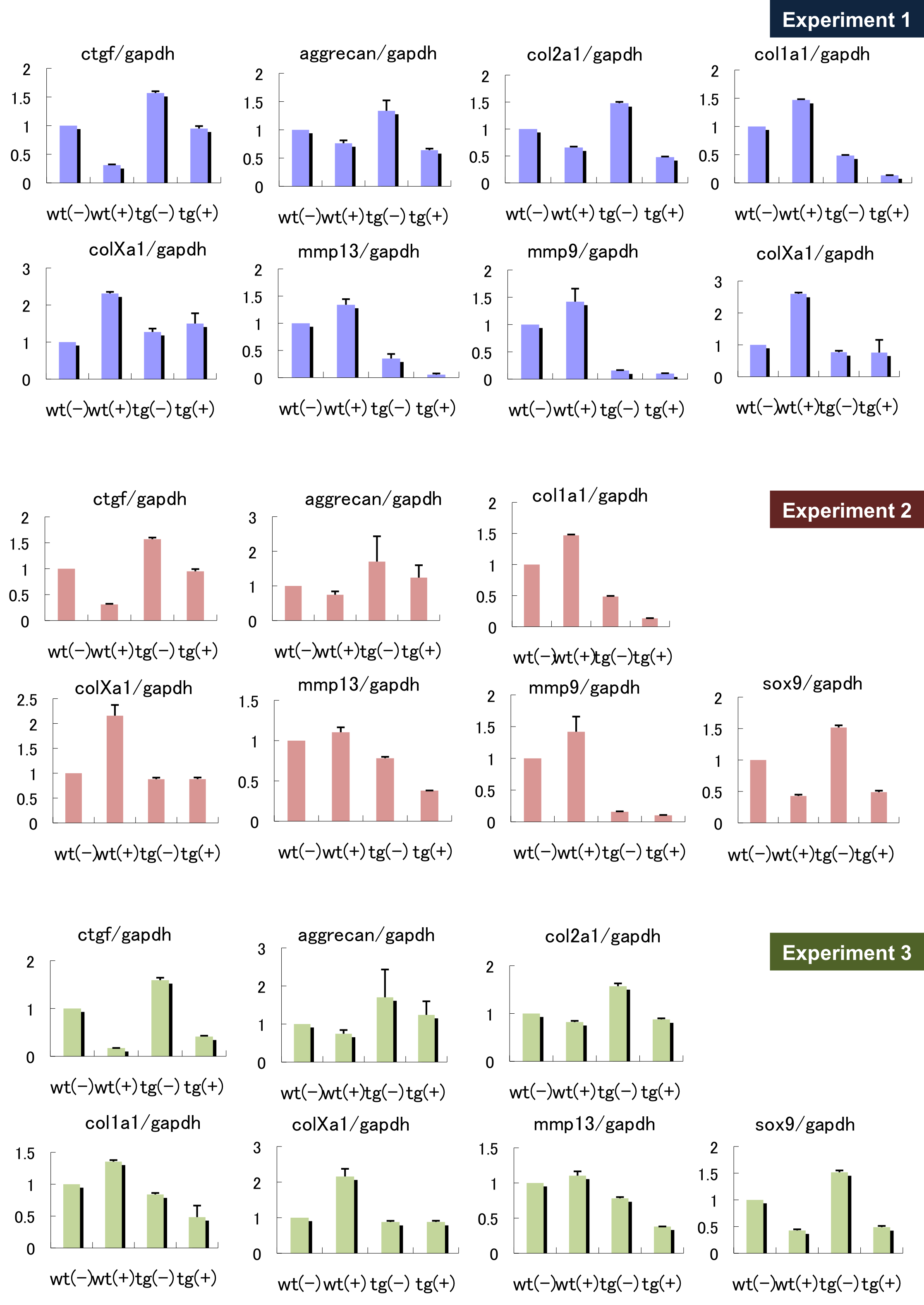

Supplement: Supporting Information S1 — Gene expression analysis of cartilage matrix constituents and proteases of epiphyseal chondrocytes from 6-day-old TG and WT mice after CTS. The experiment was done several times, and the most typical results were shown in Figure 8. (TIF) [file pone.0071156.s004.tif]
